# The Association between Intradialytic Hypertension and Metabolic Disorders in End Stage Renal Disease

**DOI:** 10.1155/2018/1681056

**Published:** 2018-04-04

**Authors:** Vaia D. Raikou, Despina Kyriaki

**Affiliations:** ^1^Department of Nephrology, Doctors' Hospital, Athens, Greece; ^2^Department of Nuclear Medicine, General Hospital “Laïko”, Athens, Greece

## Abstract

**Background:**

Intradialytic hypertension was associated with a high mortality risk. We examined the relationship between intradialytic hypertension and metabolic disorders in hemodialysis treatment patients.

**Methods:**

We studied 76 patients in online hemodiafiltration. Dialysis adequacy was defined by *Kt*/*V* for urea. Normalized protein catabolic rate (nPCR), as a marker of protein intake, was calculated. Sodium removal was determined as percent sodium removal. Metabolic acidosis was determined by serum bicarbonate less than 22 mmol/L. Interdialytic urine volume more than 100 ml was recorded. Intradialytic hypertension was defined by an increase in systolic blood pressure equal to 10 mmHg from pre- to posthemodialysis. Arterial stiffness was assessed as carotid-femoral pulse wave velocity (c-fPWV) and carotid augmentation index (AIx). Chi-square tests and logistic regression analysis were applied for intradialytic hypertension prediction.

**Results:**

Patients with intradialytic hypertension were older and had significantly lower hemoglobin, nPCR, urine output, and serum bicarbonate and significantly higher c-fPWV, though similar *Kt*/*V* for urea, than patients without intradialytic hypertension. They also had increased sodium removal and pulse pressure related to less urine output. Serum bicarbonate was inversely associated with c-fPWV (*r* = −0.377, *p* = 0.001). Chi-square test showed significant association between intradialytic hypertension and serum bicarbonate < 22 mmol/L (*x*^2^ = 5.6, *p* = 0.01), which was supported by an adjusted model.

**Conclusion:**

The intradialytic hypertension was significantly associated with metabolic disorders including malnutrition/inflammation and uncontrolled metabolic acidosis in hemodialysis treatment patients. Severe metabolic acidosis may reflect sodium imbalance and hemodynamic instability of these patients resulting in volume overload and increased vascular resistance.

## 1. Introduction

In chronic kidney disease, hypertension is influenced by both blood pressure (BP) and the progression of renal disease. In end stage renal disease (ESRD) on maintenance hemodialysis, hypertension is a main feature and more than 85% of new patients with ESRD present with hypertension [1].

Hypertension in these patients is multifactorial. Significant implicating factors include persistent hypervolemia and elevated peripheral vascular resistance. In patients with 3 hemodialysis sessions per week, blood pressure increases during the interdialytic interval according to weight gain, particularly in older patients and those with higher dry weight. The main goal of hemodialysis treatment is the control of extracellular volume (ECV), because inadequate sodium and fluid removal results in fluid overload, increased BP, and increased mortality [2]. Elevated peripheral resistance can be attributable to inappropriate activation of the sympathetic nervous system due to higher plasma angiotensin II and norepinephrine concentrations [1, 3].

The association of hypertension with adverse outcomes has been demonstrated, due mainly to its relationship with abnormalities in cardiac structure and cardiac function including left ventricular hypertrophy, diastolic dysfunction, and arterial stiffness [4–6].

However, BP shows a dynamic nature during dialysis procedures, including modest decreases, a common phenomenon, and intradialytic hypotension and hypertension, which are two special situations significantly related to an increased risk of mortality in these patients [7, 8]. It has been shown that intradialytic hypertension, which is a phenomenon where the blood pressure increases during and immediately after hemodialysis, was associated with a higher mortality risk than the most often intradialytic hypotension [9, 10].

In this study, we examined the relationship between intradialytic hypertension and metabolic disorders in permanent hemodialysis treatment patients.

## 2. Materials and Methods

### 2.1. Subjects

This is a dual-center observational cross-sectional study, which was reviewed and approved by the “Laïko, University General Hospital of Athens” and the Renal Unit of “Diagnostic and Therapeutic Center of Athens Hygeia SA” Institutional Review Board. 76 patients (47 men and 29 women, mean age: 62,2 ± 15 years) were included in the study, and they or their legal guardian provided informed oral consent prior to study enrolment.

The applied hemodialysis modality was online-predilution hemodiafiltration (onl-HDF) in all subjects. The median time in hemodiafiltration therapy was 5 years ± interquartile range 3–10.

The hemodiafiltration treatment was performed 3 times weekly with a dialysis time of 4 h per session. We used a filter of 1.5–2 m^2^ surface area by high-flux synthetic membrane, defined by an ultrafiltration coefficient > 20 ml/h [11] in all participants. We also used the same volume of replacement liquid equal to 20 liters, a blood flow of 350–400 ml/min, and a dialysate flow rate of 500–600 ml/min. A bicarbonate-based ultrapure buffer dialysis solution was used and the final concentration of bicarbonate in the dialysate was 32 mmol/L. A dialysate calcium concentration of 1.50–1.75 mmol/L and a sodium concentration of 138–145 mmol/L were applied. Dialysis dose was defined by sp*Kt*/*V*/session (single pool, *K*: dialyzer clearance; *t*: time; *V*: urea distribution volume) [12]. We excluded from the study subjects whose calculated sp*Kt*/*V*/session was less than 1.2.

We also excluded patients <18 years of age at initiation of dialysis treatment and patients with less than 6 months of follow-up or those without regular vascular hemodialysis access. Subjects with autoimmune diseases, infections, and atrial fibrillation malignancy and those with interdialytic weight gain of more than 5% of total body weight were not included in the study. Interdialytic weight gain was calculated as the mean value over the exposure period of one treatment month.

The enrolled patients were on a free regular diet and they did not apparently have interdialytic peripheral edema or extradialytic orthostatic hypotension. Nineteen of the participants were current smokers (a ratio of 25%). Twenty of the enrolled patients disposed residual renal function defined by an interdialytic urine volume of more than 100 ml (a ratio of 26.3%).

Only calcium-free phosphate binders including sevelamer carbonate, lanthanum, and/or aluminium hydroxide were prescribed in combination with vitamin D derivatives for the regulation of bone disease (a ratio of 44.7%). None of our participants was receiving NaHCO_3_ per os or warfarin therapy. All the included patients were treated by erythropoietin *α* or *β* agents.

In our data, the cause of renal failure included hypertensive nephrosclerosis at a ratio of 32.9%, chronic glomerulonephritis at a ratio of 28.9%, polycystic disease at a ratio of 11.8%, diabetic nephropathy at a ratio equal to 9.2%, and other causes at a ratio of 17.1%.

### 2.2. Blood Pressure Measurements: Definitions

Blood pressure data were considered over a treatment month, as the exposure period, which typically included 12 dialysis sessions per patient. Systolic blood pressure (SBP) was measured with the patient in the seated position using automated oscillometric devices immediately before, after, and during (at 30-minute intervals) all treatment sessions. We excluded treatments in which SBP was measured <3 times.

We defined intradialytic hypertension as an increase in SBP equal to 10 mmHg from pre- to posthemodialysis according to previous reports [7]. Participants who had an average change in SBP from pre- to immediately posthemodialysis equal to or more than 10 mmHg throughout the study period were considered to have intradialytic hypertension (*n* = 15, a ratio 19.7%).

On the other hand, the measurement of blood pressure at home was requested using an automatic sphygmomanometer Omron M4-I (Omron Co. Ltd., Kyoto, Japan). The blood pressure was doubly measured two times per day, in the morning after rising and in the evening in a fasting, calming, and resting state, and two means were recorded per day. Their average was used for statistical analysis. Mean peripheral blood pressure (MBP) was calculated as MBP = DBP + 1/3 (SBP − DBP).

We also used a 24-hour ambulatory blood pressure monitor with the Mobil-O-Graph device for verification of measurements and whether the mean blood pressure values significantly differed from home recorded values; the means of 24-hour monitoring were used for statistical analysis rather than the means by home measurements.

In accordance with the KDOQI hypertension threshold [13], the participants with mean blood pressure > 130/80 mmHg (*n* = 29, a prevalence of 38.2%) assessed by 24-hour monitoring and/or home measured according to recommended standard protocol or subjects with an individual hypertension history were considered hypertensive and every one of them was regularly receiving antihypertensive therapy including calcium channel blockers, beta-blockers, or inhibitors of angiotensin II AT1 receptors.

### 2.3. Hemodynamic Measurements

Before the midweek dialysis session, the participants rested for at least 10 minutes and hemodynamic measurements were performed. Arterial stiffness was measured as carotid-femoral pulse wave velocity (c-fPWV) and carotid augmentation index (AIx) using the SphygmoCor System® (AtCor Medical Pty. Ltd., Sydney, Australia) according to the manufacturer's specifications [14]. In each subject, two sequences of measurements were performed, and their mean was used for statistical analysis. Pulse pressure (PP) was derived.

### 2.4. Blood Collection

Blood samples were obtained just before the start of the mean weekly dialysis session in a twelve-hour fasting state from the vascular access of enrolled subjects and serum was separated and processed for various assays. At the end of the session, blood samples was drawn at 2 min after dialysis from the arterial dialysis tubing after the reduction of blood pump speed to less than 80 ml/min in order for the dose of dialysis session to be calculated using sp *Kt*/*V* for urea [14]. The mean of 12 calculations for sp *Kt*/*V* for urea per dialysis session during a treatment month was used for statistical analysis.

In each subject, four sequences of samples (every week within the exposure period) were obtained for the serum bicarbonate measurements, and their average was used for statistical analysis. We paid attention thus to the low serum bicarbonate level to be combined with low arterial pH (acidemia) and decreased PCO_2_, which determines the metabolic acidosis presence, rather than respiratory alkalosis, which is another clinical condition that causes decreased bicarbonate level, but without acidemia [15].

### 2.5. Laboratory Measurements

Albumin, calcium (Ca) corrected for the albumin levels, phosphate (P), high-density lipoproteins (HDL), and low-density lipoproteins (LDL) were measured by photometric biochemical analysis (MINDRAY BS-200, Diamond Diagnostics, USA). The ratios of LDL/HDL and Ca × P products were calculated. Sodium (Na^+^) levels both at the start and at the end of the treatment session were also measured by biochemical analysis. Sodium removal was determined as percent sodium removal (PSR) using the following formula: (Na^+^ pre − Na^+^ post/Na^+^ pre) × 100.

The evaluation of sodium removal was used for the estimation of extracellular volume (ECV) assuming that the change in body weight during a dialysis session represents the change in extracellular volume due to ultrafiltration, in combination with clinical characteristics related to an incorrect dry body weight including the presence of interdialytic peripheral edema, interdialytic orthostatic hypotension, or extradialytic uncontrolled blood pressure.

Hematological analyzer (Sysmex, xt-4000i, Roche, Germany) was used for hemoglobin (Hb).

The concentration of intact parathormone (i-PTH) was measured by radioimmunoassay (CIS Bio International, France).

High sensitivity C-reactive protein (hsCRP) serum concentrations were measured using enzyme-linked immunosorbent assay (ΕLISA, Immundiagnostik AG, Germany) according to the manufacturer's specifications.

Metabolic acidosis was defined by serum bicarbonate concentrations less than 22.0 mmol/L, which were measured in gas machine (Roche, cobas b 121) by an electrode-based method taking care of the blood specimens [16].

Normalized protein catabolic rate for dry body mass (nPCR) was calculated from the urea generation rate [17]. Body mass index (BMI) was obtained from height and postdialysis body weight.

## 3. Data Analysis

Data were analyzed using SPSS 15.0 statistical package for Windows (SPSS Inc., Chicago, Illinois) and expressed as mean ± standard deviation or as median value (interquartile range) for data that showed skewed distribution. Differences between mean values were assessed using unpaired *t*-test for two groups and data that showed skewed distributions were compared with Mann–Whitney *U* test.

Correlations between variables were defined by Spearman's coefficient and the relationships between categorical variables were defined by chi-square tests. *p* values less than 0.05 were considered significant. We built a model using logistic regression analysis by enter method in order to define the risk factors which could impact the installation of intradialytic hypertension in our data using traditional and specific variables for these patients.

## 4. Results

In Table 1, the differences between the groups of patients with (*n* = 15) and without (*n* = 61) intradialytic hypertension are shown.

We observed that the patients with intradialytic hypertension were older and had significantly lower Hb, nPCR, urine output, and bicarbonate serum concentrations than patients without intradialytic hypertension. However, they had significantly higher c-fPWV and AIx in comparison with patients without intradialytic hypertension. They also had higher sodium removal, Ca × P products, PP, dialysis vintage, and hsCRP, but lower i-PTH than patients without intradialytic hypertension. Both groups of patients had similar BMI, albumin, dialysis sufficiency, and interdialytic weight gain.

**Figure 1:**
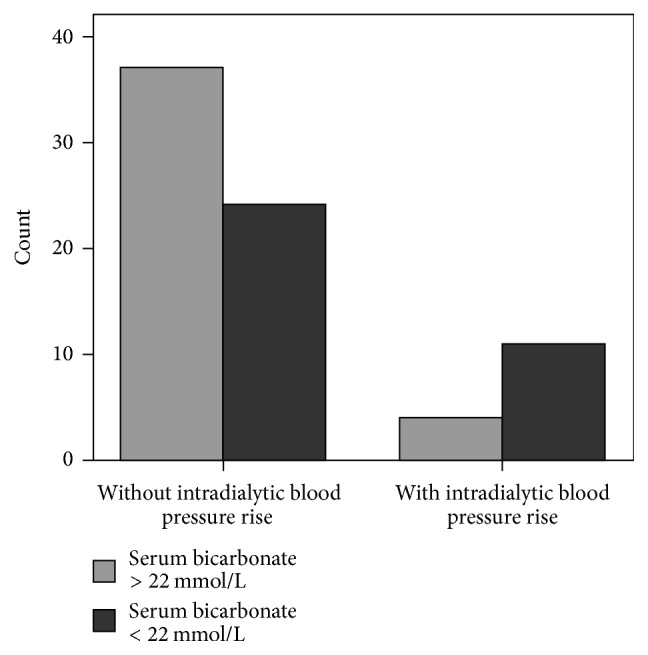
Bar chart for the association between intradialytic hypertension and metabolic acidosis state defined by serum bicarbonate less than 22 mmol/L (*x*^2^ = 5.6, *p* = 0.01).

**Figure 2:**
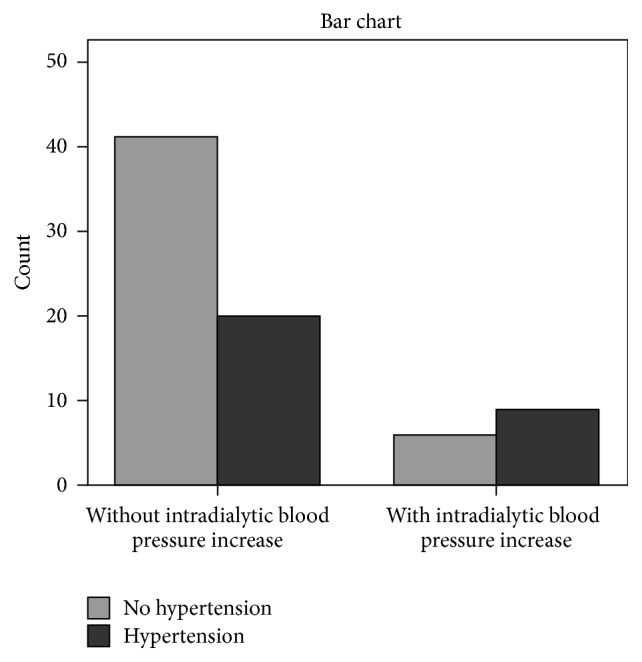
The prevalence of intradialytic hypertension in participants with extradialytic hypertension (*x*^2^ = 4.2, *p* = 0.04).

Chi-square tests showed significant association between the prevalence of intradialytic hypertension and both serum bicarbonate concentrations < 22 mmol/L and extradialytic hypertension (*x*^2^ = 5.6, *p* = 0.01 and *x*^2^ = 4.2, *p* = 0.04, resp.) (Figures 1 and 2). The association between intradialytic hypertension and preservation or not of residual renal function defined by urine output was found to be nonsignificant.

In the built adjusted model for the factors which could impact the manifestation of intradialytic hypertension, we found the old age, the bigger dialysis vintage, the elevated sodium removal, and the lower serum bicarbonate to be significant risk factors adjusting to diabetes mellitus, extradialytic hypertension, Hb, nPCR, hsCRP, and dialysis sufficiency defined by sp*Kt*/*V* for urea (Table 2).

**Figure 3:**
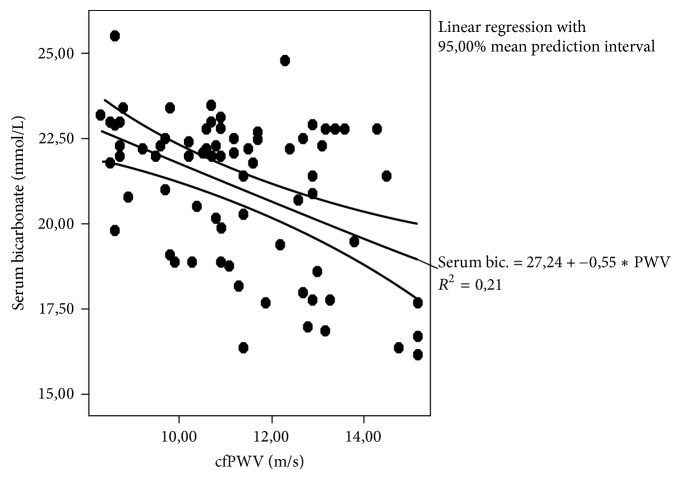
The correlation between carotid-femoral pulse wave velocity (c-fPWV) and serum bicarbonate concentrations (*r* = −0.377, *p* = 0.001).

**Figure 4:**
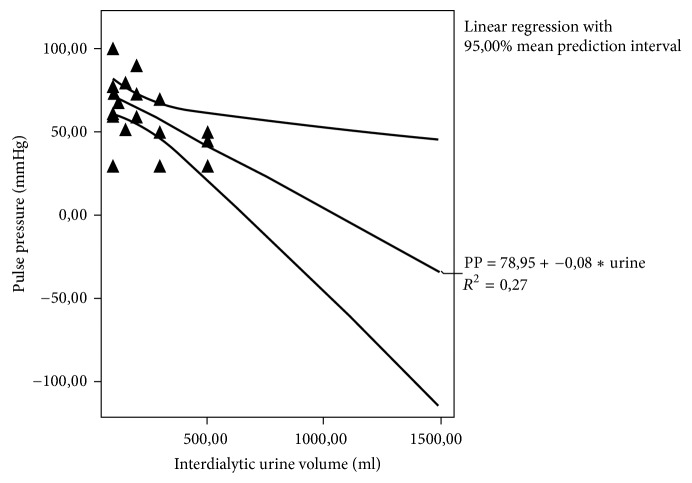
The correlation between pulse pressure and interdialytic urine volume (*r* = −0.481, *p* = 0.03).

Bivariate correlations showed significant association between c-fPWV and hsCRP, i-PTH, and serum bicarbonate (*r* = 0.399, *p* = 0.001; *r* = 0.361, *p* = 0.02; and *r* = −0.377, *p* = 0.001, Figure 3, resp.). We also did note significantly inverse association between urine output and pulse pressure (*r* = −0.481, *p* = 0.03, Figure 4).

## 5. Discussion

The variability of BP during hemodialysis treatment, which is defined by either intradialytic hypotension or intradialytic hypertension, may be attributed in hemodynamic abnormalities or/and in a paradoxical response to the dialysis procedure in a subset of hemodialysis patients [8]. Although intradialytic hypotension occurs more often compared to intradialytic hypertension, it has been reported that intradialytic hypertension confers a higher mortality risk than hypotension [9]. Recently, it has been shown that frequent intradialytic hypertension was associated with increased 30-day morbidity and mortality considering the intradialytic hypertension as a short-term risk marker additionally to long-term mortality [18].

The prevalence of intradialytic hypertension has been described in 5–20% of hemodialysis treatments [9, 18]. In our data, the prevalence of this phenomenon reached 19.7%.

The history of hypertension is the cause for the phenomenon of intradialytic hypertension in a number of dialysis patients [19, 20]. Indeed, in this study, we noted a significant unadjusted association between intradialytic BP rise and extradialytic hypertension. The removal of antihypertensive medications during hemodialysis treatment may be also a contributor to intradialytic BP rise. However, a previous study demonstrated that the antihypertensive number, class, and dialyzability status were not significantly associated with intradialytic BP variability [21].

The pathophysiology of intradialytic hypertension is poorly explained, even though the mechanisms and management of this phenomenon have been investigated in numerous studies over the past few years. A conjunction among positive sodium balance, volume overload, activation of the renin-angiotensin aldosterone (RAAS) and sympathetic nervous system, endothelial cell dysfunction, erythropoiesis-stimulating agents, and bone mineral disease abnormalities has been suggested.

Hypervolemia is a well-recognized risk factor for hypertension among dialysis patients. Patients with intradialytic hypertension have been found to be more chronically volume-overloaded than other hemodialysis patients, although they typically may have small interdialytic weight gain and clinically do not appear to be volume-overloaded [9, 22]. Indeed, in this study, patients with intradialytic hypertension had no apparent peripheral edema or uncontrolled extradialytic hypertension and they had similar interdialytic weight gain to those without intradialytic hypertension. However, they had significantly lower urine output in conjunction with higher sodium removal, higher PP, and increased arterial stiffness markers including c-fPWV and AIx in comparison to the patients without intradialytic hypertension. We also did note significantly inverse association between urine volume and PP.

To obtain a good BP control in dialysis patients, we must define the correct dry weight and individualize the adequate sodium concentration in dialysate, thus to achieve a zero intradialytic sodium balance [23]. Elevated sodium removal may be due to our trying to reach a dry weight lower than the correct one, which may have, as a consequence, intradialytic hypotensive episodes and muscle cramps, which can lead to the need for an increase in dialysate sodium concentration, thirst, and eventually greater interdialytic weight gain causing as a final result an ECV increase and hypertension [24]. Such a phenomenon is particularly exciting when a lower urine volume is combined, such as in our subjects with intradialytic hypertension. Moreover, in our previous study, PP was found to be influenced by volume overload rather than by arterial stiffness [25] and this finding may be confirmed by the found significantly inverse association between urine output and PP in present study. In the meantime, it has been already reported that fluid overload plays an important role in the development of arterial stiffness in dialysis patients and PWV varies during dialysis due to alterations in hydration status [26, 27]. All the above could suggest that our participants with intradialytic hypertension were fluid-overloaded even though they apparently were not.

On the other hand, although a causal role of volume overload has not been established, factors beyond volume overload are important mediators of intradialytic hypertension in many patients [22]. A link between volume overload and endothelial dysfunction markers has been suggested, which may be also stimulated by a greater sodium concentration in dialysate [28]. Recent investigations have established endothelial cell dysfunction as a key mediator in intradialytic hypertension and elevated vascular resistance, which remains the main driving force for BP increases [7, 9, 10]. Endothelial cells contribute to BP homeostasis by producing and releasing factors such as nitric oxide, a smooth muscle vasodilator, and endothelin-1, a vasoconstrictor. Among patients with intradialytic hypertension, studies have demonstrated that endothelin-1 levels rise during dialysis while systemic nitric oxide levels remain inappropriately low [29, 30]. Erythropoietin stimulating agents may contribute to intradialytic hypertension via this mechanism [31]. However, it has been suggested that mediators other than endothelin-1 may be responsible for increased intradialytic vascular resistance [9]. Our recent previous study showed that the loss of residual renal function related to fluid overload was associated with cardiovascular outcomes in dialysis patients, due additionally to the coexistence of increased levels of monocyte chemoattractant protein-1 (MCP-1), a chemokine, which may reflect a progressive inflammation/oxidative stress condition [14].

Clinical characteristics associated with intradialytic BP rise include older age, lower body weight, lower serum creatinine and albumin, and utilization of more antihypertensive medications [32, 33]. Lower albumin and predialysis urea nitrogen levels may contribute to small reductions in osmolarity during dialysis and this prevents the blood pressure from falling. In this study, subjects with intradialytic hypertension were older and had significantly lower hemoglobin, lower nPCR as a marker of protein intake and malnutrition, and lower i-PTH, although they had higher hsCRP resulting in the cooccurrence of malnutrition, inflammation, and atherosclerosis (MIA syndrome), in comparison to the patients without intradialytic hypertension. In agreement, previously, it has been reported that low, rather than high, i-PTH was associated with inflammation and oxidative stress, as a result of MIA syndrome [34].

Malnutrition and anemia have already been reported as specific cardiovascular risk factors for dialysis patients and each of the MIA syndrome components worsens the survival of these patients [35, 36]. Malnutrition may be related to metabolic acidosis due to increased protein catabolism, decreased protein synthesis, endocrine abnormalities, and inflammation among dialysis patients [37]. Metabolic acidosis defined by low serum bicarbonate (<22 mmol/L) is a common condition in end stage renal disease patients resulting in inflammatory stimulation, lipids oxidation, and oxidative stress [38, 39]. Maintenance dialysis therapies are often unable to completely correct the base deficit. Previously, the association of uremic acidosis with arterial pressure has been reported in hypertensive patients [40].

In this study, we observed that our subjects with intradialytic BP rise had significantly lower serum bicarbonate levels than the other patients. This finding cannot be attributed to reduced dialysis treatment adequacy or other treatment related conditions, because both groups of patients had obtained a similar sp*Kt*/*V* for urea and they followed the same therapy rules. However, the older age, the cooccurrence of malnutrition/inflammation, the bigger dialysis vintage, and the lower urine output may contribute to a higher metabolic acidosis state in intradialytic hypertension patients. The unadjusted association between uncorrected metabolic acidosis and intradialytic hypertension was found to be significant in our data, as a confirming finding.

Interestingly, our subjects with intradialytic hypertension and higher metabolic acidosis state had simultaneously less urine output and increased arterial stiffness markers, which also reflect fluid overload apart from vascular injury, as it was explained above. Moreover, we did note significantly inverse association between c-fPWV and serum bicarbonate concentrations. In support, our adjusted model discovered that the low serum bicarbonate in conjunction with high sodium removal, older age, and a long dialysis time were important risk factors for manifested intradialytic hypertension adjusting for diabetes mellitus, dialysis adequacy, extradialytic hypertension, and MIA syndrome components. Particularly, the high sodium removal increased the risk for intradialytic BP rise to 1.9-fold (1.09–3.2) in our data.

These findings could suggest that uncorrected metabolic acidosis results in intradialytic BP rise in hemodialysis patients, due to its relationship with sodium imbalance and volume overload in these patients, even if clinically nonapparent, apart from its role in the increased ionized plasma calcium, which is already associated with hypertension [41]. Previously, we and others showed that patients with severe acidosis had less diuresis and they presented with important fluid overload and cardiovascular morbidity, in agreement with the findings of the present study [15, 42]. Furthermore, we could support that multiple products, other than endothelin-1, derived by severe acidosis, possibly contribute to endothelial dysfunction, resulting in elevated vascular resistance and intradialytic hypertension.

## 6. Conclusion

The manifestation of intradialytic hypertension was significantly associated with metabolic disorders including malnutrition/inflammation and uncontrolled metabolic acidosis in permanent hemodialysis treatment patients. Severe metabolic acidosis may reflect sodium imbalance and hemodynamic instability of these patients resulting in volume overload, despite being clinically nonapparent and despite the increased vascular resistance.

## Figures and Tables

**Table 1:** Differences between groups of patients according to the manifestation of intradialytic hypertension in a total of 76 subjects in hemodiafiltration (^*∗*^*p* ≤ 0.05).

Characteristic	Patients with intradialytic hypertension(*n* = 15) *Mean ± SD/mean rank*	Patients without intradialytic hypertension(*n* = 61) *Mean ± SD/mean rank*	*p value*
Age (years)	70.2 ± 14.3^*∗*^	60.3 ± 14.6	*0.02*
Dialysis vintage (years)	/45.2	/36.9	0.2
*Kt*/*V* for urea	/40.9	/37.9	0.6
nPCR (g/Kg/day)	2.1 ± 0.6^*∗*^	2.4 ± 0.5	*0.03*
Urine volume (ml/day)	100.5 ± 0^*∗*^	238.7 ± 147.7	*0.002*
BMI (Kg/m^2^)	23.9 ± 3.7	24.6 ± 2.8	0.4
Serum bicarbonate (mmol/L)	/24.03^*∗*^	/42.06	*0.005*
i-PTH (pg/ml)	/35.7	/39.2	0.6
Calcium corrected to albumin (mg/dl)	9.7 ± 0.7	9.4 ± 0.6	0.2
P (mg/dl)	5.5 ± 1.8	5.4 ± 1.9	0.8
Ca × P products	53.7 ± 17.1	50.9 ± 18.1	0.6
Interdialytic weight gain (liters)	2.05 ± 0.9	2.2 ± 0.9	0.5
Hb (gr/dl)	11.3 ± 1.6^*∗*^	12.5 ± 1.2	*0.05*
Albumin (gr/dl)	/35.6	/39.2	0.5
LDL/HDL	2.06 ± 0.8	2.4 ± 0.9	0.1
c-fPWV (m/s)	12.3 ± 2.03^*∗*^	11.08 ± 1.7	*0.02*
Augmentation index (AIx, %)	26 ± 2.2^*∗*^	23.7 ± 1.9	*0.001*
Pulse pressure (PP, mmHg)	65.2 ± 23.8	56.5 ± 17.8	0.1
Percent sodium removal (PSR, %)	/46.8	/36.5	0.08
hsCRP (mg/L)	8.3 ± 5.6	7.9 ± 5.9	0.8

**Table 2:** Logistic regression model by enter method showing risk factors for demonstration of intradialytic hypertension in our data.

Characteristic	*p* value	Odds ratio	Confidence interval
Age	**0.007**	**1.2**	**1.04**–**1.3**
Diabetes mellitus	0.9	1.06	0.006–183.8
Extradialytic hypertension	0.7	1.4	0.2–10.1
Dialysis vintage	**0.02**	**1.3**	**1.03**–**1.6**
Hemoglobin	0.6	0.8	0.3–2.0
nPCR	0.1	0.2	0.02–1.5
hsCRP	0.1	0.9	0.7–1.04
*Kt*/*V* for urea	0.2	0.02	0.0–13.5
Percent sodium removal	**0.02**	**1.9**	**1.09**–**3.2**
Serum bicarbonate	**0.006**	**0.5**	**0.3**–**0.8**

## Additional Points

*Limitations*. The main limitation of this study is the small number of included patients in combination with its cross-sectional nature. The measurement of the total serum anions for actual metabolic acidosis calculation was also unavailable in this study.
